# Effects of costimulation on intrahepatic immunopathogenesis in patients with chronic HBV infection

**DOI:** 10.1007/s00011-013-0691-3

**Published:** 2013-12-14

**Authors:** Bei Zhong, Mao Ping Huang, Guo Qing Yin, Xiang Gao

**Affiliations:** 1 The Affiliated Qingyuan Hospital, Jinan University Medical School, Yinquannan Road, Qingyuan, 511500 Guangdong China; 2 Department of Infectious Disease, Nanjing Zhong-Da Hospital, Southeast University School of Medicine, 87 Ding jia Qiao, Nanjing, 210009 Jiangsu China; 3 The Second Affiliated Hospital, Southeast University School of Medicine, 1-1 Zhongfu Road, Nanjing, 210003 Jiangsu China; 4 MOE Key Laboratory of Model Animal for Disease Study, Model Animal Research Center, Nanjing University, Xuefu Road, Pukou District, Nanjing, 210061 Jiangsu China

**Keywords:** Chronic hepatitis B infection, Costimulatory molecule, Intrahepatic immunopathophysiology, Immune tolerance

## Abstract

**Objective:**

Chronic HBV infection can lead to “immune tolerance” in asymptomatic carriers (ACs), “immune injury” in active chronic hepatitis (ACH) patients or “immune abnormality” in cirrhosis (Cir) and hepatocellular carcinoma (HCC) patients. Previous investigations reported that chronic hepatitis presented abnormal expression of costimulatory molecules. We investigated the costimulation profile in the liver of ACs and patients with ACH, Cir and HCC.

**Methods:**

Patients with ACH, Cir and HCC, ACs and normal controls were recruited into the present study. The costimulation profiles and cytokines in the liver of patients were investigated by Western blotting, immunohistochemistry and real-time quantitative PCR. Correlations between serum alanime aminotransferase (ALT) levels, necroinflammation scores, cytokines and costimulatory proteins were assessed.

**Results:**

The ACs presented decreased inflammatory and increased inhibitory costimulation, which was negatively correlated with inflammatory costimulatory proteins and ALT, whereas the ACH patients exhibited increased inflammatory costimulation and decreased inhibitory costimulation, which was correlated with increased ALT. The Cir patients showed both increased inhibitory and inflammatory costimulation. The HCC patients exhibited both decreased inhibitory and inflammatory costimulation.

**Conclusion:**

Costimulation participates in intrahepatic immune responses, and plays important roles in immune tolerance, immune injury and immune abnormalities in patients with chronic HBV infection.

**Electronic supplementary material:**

The online version of this article (doi:10.1007/s00011-013-0691-3) contains supplementary material, which is available to authorized users.

## Introduction

Infection with hepatitis B virus (HBV) is a common cause of chronic hepatitis. Approximately 350 million people worldwide are chronically infected with HBV [1, 2]. Because HBV is not usually cytopathogenic by itself, chronic HBV infection (CHB) is a dynamic state of interaction between the virus, hepatocytes and the host’s immune system. The natural course of CHB is generally divided into three phases, namely the immune-tolerant phase, the immune clearance phase and the residual or inactive phase. In the immune-tolerant phase, subjects with HBV infection are known as asymptomatic carriers (ACs), presenting persistent immune tolerance against HBV antigens but no evidence of hepatitis [2]. As the immune-tolerant phase changes to the immune clearance phase, patients suffer from intrahepatic inflammatory activity and hepatitis flare. However, these inflammatory responses do not clear HBV in hepatocytes. Persistent HBV reproduction and inflammatory reaction in the liver result in progressive liver disease. Patients with progressive liver diseases present a spectrum of diseases ranging from active chronic hepatitis (ACH), through cirrhosis (Cir) to hepatocellular carcinoma (HCC). ACH patients exhibit “immune injury” and display active hepatitis, and Cir or HCC patients present “immune abnormalities” [1, 2]. Finding the distinct immune status and investigating the immunopathophysiological differences between immune tolerance, immune injury and immune abnormalities may clarify the immune mechanism of CHB.

The innate and adaptive immune responses induce liver diseases in CHB patients. Both immune regulatory factors and immune cells participate in the clearing of virus components, such as costimulatory factors, cytokines, dendritic cells (DCs), HBV-specific effector T cells, regulatory T cells, non-virus-specific lymphocytes, natural killer cells and B cells. However, the immune responses are extremely complex and only partially understood [3]. Costimulatory proteins, acting as signal peptides, contribute to signal transference among immune cells, and are functional markers of immune cells. When clearing a virus, the viral antigenic peptides are first processed by antigen-presenting cells, mainly DCs, and are bound by MHC II or MHC I complexes in DCs. This binding contributes to DC maturation. The matured DCs are marked by CD83^+^, CD80^+^ and CD86^+^. Subsequently, clonally distributed T-cell antigen receptors on naive T cells capture the viral antigenic peptide–MHC II or MHC I complexes on matured DCs, and CD28 on T cells binds with CD80/CD86 on DCs. This process induces expansion and differentiation of clonal T cells and translates naive T cells into functional T cells. In contrast, cytotoxic T-lymphocyte antigen 4 (CTLA-4) on T cells, acting as an inhibitory factor, negatively regulates the CD28/CD80/CD86 pathway and inhibits T-cell activity. Finally, the functional T cells activate B cells and other immune cells, resulting in high expression of CD40 and intercellular adhesion molecule-1 (ICAM-1) and inducing secretion of inflammatory cytokines [4–9]. In short, costimulation regulates the immune responses.

Various authors have demonstrated that expression of costimulatory molecules is abnormal in chronic hepatic diseases and viral hepatitis and that abnormal costimulation contributes to immunopathogenesis in liver diseases [4–9]. Nevertheless, their studies have obvious limitations. Firstly, they investigated CHB immunopathophysiology on costimulation signal pathways using animal experiments [4, 6]. However, the resulting model of HBV infection has not reproduced the whole disease spectrum of humans, including ACs and ACH, Cir and HCC patients. CHB immunopathophysiology must be demonstrated in human liver obtained from the whole disease spectrum. Secondly, the immune tolerance in ACs is a distinct immune state, and its immunopathophysiology in peripheral blood is only partly understood [10], but the intrahepatic costimulation profile has not been investigated. Finally, the liver is an immune-tolerant organ, in which apoptosis and degeneration of functional immune cells take place, resulting in intrahepatic immune suppression [11]. In previous papers, different states of immunopathophysiology between the liver and peripheral blood were defined as “immune compartmentalization”, which was found in investigations on animals and humans [11–14]. Costimulatory factor mRNA and costimulation-positive cells were detected in liver and peripheral blood of patients [15, 16], but costimulatory protein in human liver was not studied. In fact, the profile of costimulatory proteins in the liver of patients, and not their peripheral blood, exhibits the actual immunopathophysiology of CHB.

We hypothesized that intrahepatic costimulation in ACs and in ACH, Cir and HCC patients was different and that the distinct costimulation profiles might contribute to the intrahepatic immunopathophysiology. Therefore, it was necessary to directly detect costimulatory proteins in the liver of patients.

## Materials and methods

### Liver samples and patients

Patients and normal controls, namely ACs, ACH, Cir and HCC patients and healthy donors (HDs), were recruited from January 7, 2002 to June 5, 2006 at four hospitals in Jiangsu Province, China: the Second Affiliated Hospital, Southeast University Medical School; the Surgery Department of the First People’s Hospital of Huai’an City; the Liver Transplantation Center of the First Affiliated Hospital, Nanjing Medical University; and the Surgery Department of the People’s Hospital of Tai’xing City. AC, ACH, Cir and HCC were defined according to the criteria reported by Lok et al. [17]. The liver tissues were obtained from liver transplantations, surgical operations and biopsies. This research was carried out in accordance with the Declaration of Helsinki (2000) of the World Medical Association. The plan was approved by the Health Office of Jiangsu provincial government, the Health Office of Nanjing municipal government and all the hospital ethics committees. All the patients or the immediate family members of liver donors signed informed consent documents.

Negative HBeAg patients generally have a predominance of precore and/or basal core promoter HBV mutations that are hence either unable to express or express low levels of HBeAg. These HBV mutations might activate various immune responses in the host, and eventually result in fulminant hepatitis [30]. In order to avoid the influence of precore and the basal core promoter mutations, negative HBeAg patients were excluded from the present study.

### Antibodies and reagents

Antibodies for CD83 (H-198, SC-20083) and ICAM-1 (H-108, SC-7891) and positive proteins for CD80 (separated Ramos cell) and CD86 (separated Jurkat cell) were purchased from Santa Cruz Biotechnology Inc. Antibodies for CD40 (BWC02, AF632), CD28 (ADS013091, AF-342-PB), CTLA-4 (AF-386-BP), CD80 (AAE02, AF140), CD86 (AAE01, AF-141-NA) and β-actin and standard proteins, namely recombinant human CD80 protein (140-B1) and recombinant human CD86 protein (140-B2), were purchased from R&D Inc. Pre-staining protein marker (P7708S) was obtained from New England BioLabs, (USA), and ABC immunochemical kits from Vector Laboratories Inc. (Burlingame, CA, USA). All other reagents were bought from Sigma.

### Western blotting of costimulatory proteins in liver

The relative quantity of costimulatory proteins and of DC marker proteins in liver were detected by Western blotting as described by Zhong et al. [18]. Livers were homogenized in lysis buffer containing 50 mm Tris HCl (pH 6.8), 2 % sodium dodecyl sulfate, 10 % glycerol, 50 mm dithiothreitol and 0.1 % bromophenol blue, and were diluted to 1 mg total protein/ml. Samples and lysate of liver tissue were resolved in 8 % SDS-PAGE. Proteins were blotted onto nitrocellulose membrane and incubated with primary antibody and secondary antibody. Immune complexes were visualized using an enhanced chemiluminescence substrate by ECL^TM^ (Amersham Pharmacia Biotech, Arlington Heights, IL, USA). The duration of autoradiography in each protein is shown in Table 1, and the images on the X-ray membrane were scanned. The relative quantity of protein was normalized to the protein quantity for each sample using β-actin protein as an internal standard.

**Table 1:** Working parameters for Western blotting

Item	Primary antibody dilution rate	Second antibody dilution rate	Duration of autoradiography (min)
CD80	1:100 (goat anti-human CD80 antibody)	1:2,500 (mouse anti-goat IG antibody)	300
CD86	1:100 (goat anti-human CD86 antibody)	1:7,000 (mouse anti-goat IG antibody)	300
CD83	1:100 (rabbit anti-human CD83 antibody)	1:8,000 (mouse anti-rabbit IG antibody)	300
CD28	1:100 (goat anti-human CD86 antibody)	1:5,000 (mouse anti-goat IG antibody)	300
CTLA4	1:100 (goat anti-human CD86 antibody)	1:5,000 (mouse anti-goat IG antibody)	360
CD40	1:100 (goat anti-human CD86 antibody)	1:2,500 (mouse anti-goat IG antibody)	300
ICAM-1	1:500 (rabbit anti-human CD83 antibody)	1:5,000 (mouse anti-rabbit IG antibody)	60
β-Actin	1:5,000 (mouse anti-human β-actin antibody)	1:15,000 (goat anti-mouse IG antibody)	10

Costimulatory protein are present in low concentrations. The duration of autoradiography for each protein was determined at different durations, viz. 10, 20, 40, 60, 120, 180, 240, 300, 360 and 420 min, with the optimal duration being used for each specific protein

Costimulatory proteins are membrane proteins on immune cells, and their content in tissue is very low. Therefore, the common technique of Western blotting is not useful for detecting costimulatory proteins in tissue. In the preliminary experiment for each protein, the liver tissue obtained from a Cir patient was firstly used to determine the optimal antibody dilution rate. The duration of autoradiography for each protein was then determined at different durations, viz. 10, 20, 40, 60, 120, 180, 240, 300, 360 and 420 min, with the optimal duration being used for each specific protein. The optimal parameters are shown in Table 1.

### Immunohistochemistry and histology

Histological sections of liver stained with hematoxylin and eosin (H&E) azure and with immunohistochemical stains of CD80, CD86, CD83, CD28, CD40, CTLA-4 and ICAM-1 were observed by light microscopy. The liver specimens were fixed in 4 % neutral buffered formalin, and embedded in paraffin. Sections 6 μm thick were prepared, deparaffined, and rinsed three times with 0.01 M PBS (pH 7.4). The sections were rehydrated and incubated with 1 % trypsinase (Sigma) for 20 min at 37 °C in order to retrieve antigen. They were then treated with 0.3 % H_2_O_2_ for 15 min to eliminate endogenous peroxidase. After blocking with nonspecific staining, the sections were incubated with primary antibody. The secondary antibody coupled to horseradish peroxidase was incubated at room temperature for 30 min, and then processed using the ABC kit of immunoperoxidase stains. Finally, these sections were counterstained with hematoxylin. The negative controls were performed by omitting the first antibody.

The necroinflammatory lesions and fibrosis were graded by the Ishak modified HAI system [19]. The maximum possible score in this system for grading of necroinflammation was 18, and for fibrosis was 6. The distribution of costimulatory proteins in liver was revealed by immunohistochemical staining.

### Real-time quantitative PCR of cytokine mRNA in liver

Real-time quantitative PCR and data analysis were performed as described by Zhong et al. [18]. Total RNA in the liver was extracted using an RNeasy mini kit (Generay Biotech Co., Shanghai, China). One μg of total RNA was reverse transcribed in a 20 μl volume using an RT-PCR kit (Promega Corporation, USA) according to the manufacturer’s instruction.

The primer sets were predesigned by Applied Biosystems. The primer sequences and annealing temperatures are shown in Table 2; and the 45-cycle PCR was performed for all items. The cytokine mRNA was normalized to RNA loading for each sample using β-actin mRNA as an internal standard. The relative mRNA quantity, including interferon (IFN)-γ, interleukin (IL)-6, IL-18 and IL-10 were detected using Engine Option^TM^ (MJReseach, USA) by SYBR Green I.

**Table 2:** PCR primer sequences and annealing temperature for detecting items

Cytokine	Sequence	Annealing temperature (°C)
IFN-γ	F 5′-GGAGACCATCAAGGAAGACAT-3′	59
	R 5′-GCGACAGTTCAGCCATCAC-3′	
IL-6	F 5′-AAGCCAGAGCTGTGCAGATGAGTA-3′	64
	R 5′-TGTCCTGCAGCCACTGGTTC-3′	
IL-10	F 5′-GAGATGCCTTCAGCAGAGTGAAGA-3′	64
	R 5′-AGGCTTGGCAACCCAGGTAAC-3′	
IL-18	F 5′-GCCTGGACAGTCAGCAAGGA-3′	60.5
	R 5′-TCTACTGGTTCAGCAGCCATCTTTA-3′	
β-actin	F 5′-TATCCTGGCTGTGCTATCCC-3′	59
	R 5′-CCATCTCTTGCTCGAAGTCC-3′	

### Statistical analysis

Data are given as means (minimal–maximal). Clinical and immunological parameters were compared by the Kruskal–Wallis test, Nemenyi test and *M* test (Friedman). Correlations between ALT, necroinflammatory scores, cytokine mRNA and costimulatory proteins were assessed using Spearman’s rank correlation coefficient. *p* values < 0.05 were considered significant.

## Results

### No evidence of hepatitis in ACs, but various characteristics of liver disease in ACH, Cir and HCC patients

The patients’ characteristics are shown in Table 3. The ACs presented high viral loads with positive HBeAg but normal ALT, whereas ACH patients exhibited increased ALT. Cir and HCC patients showed abnormal or normal ALT. By histological observation, ACs exhibited no evidence of hepatitis in liver or spotty lytic necrosis and apoptosis in lobules (necroinflammatory and fibrosis scores N 0–1 and F 0–1, respectively), similar to HDs; ACH patients showed severe necroinflammation and slight fibrosis (N 12–18 and F 1–2); and Cir patients presented mild necroinflammation and definite fibrosis (N 5–9 and F 6). However, HCC patients displayed slight necroinflammation and fibrosis in non-tumor tissue, namely focal lytic necrosis, focal inflammation, piecemeal necrosis or slight fibrosis (N 2–5 and F 0–2). The data in Table 3 show that there was no evidence of hepatitis in ACs but various characteristics of liver disease in ACH, Cir and HCC patients.

**Table 3:** Patient characteristics

Groups	Age (years)/sex	Diagnosis	HbsAg: 0–1.0 S/CO	HbeAg: 0–0.28 PEIU/ml	HBcAb: 1–3 S/CO	HBV-DNA: <2.70 copies/ml (log)	TB: 5.1-19 μmol/L	DB: 1.7-6.8 μmol/L	ALT: 5-40 u/L	Histology: necroinflammation score (N 0–18) and fibrosis score (F 0–6)	Liver specimen obtained from:
HD 1	55/M	Donor liver from liver transplant	Neg	Neg	Neg	<2.70	Normal (Nor)	Nor	Nor	N 1 and F 0	Surgical operation
HD 2	47/M	Donor liver from liver transplant	Neg	Neg	Neg	<2.70	Nor	Nor	Nor	N 1 and F 0	Surgical operation
HD 3	39/M	Donor liver from liver transplant	Neg	Neg	Neg	<2.70	Nor	Nor	Nor	N 0 and F 0	Surgical operation
HD 4	27/F	Donor liver from liver transplant	Neg	Neg	Neg	<2.70	Nor	Nor	Nor	N 0 and F 0	Surgical operation
HD 5	35/M	Donor liver from liver transplant	Neg	Neg	Neg	<2.70	Nor	Nor	Nor	N 1 and F 0	Surgical operation
HD 6	32/M	Donor liver from spleen trauma	Neg	Neg	Neg	<2.70	Nor	Nor	Nor	N 0 and F 0	Surgical operation
AC 1	51/F	Gastric ulcer	102.67	13.318	0.067	5.43	19.0	7.3	35.7	N 1 and F 1	Surgical operation
AC 2	33/M	Gastric perforation	136.73	384.072	0.104	8.08	15.7	6.8	28.5	N 0 and F 0	Surgical operation
AC 3	38/M	Spleen trauma	79.42	459.150	0.166	6.91	20.1	5.3	39.8	N 1 and F 0	Surgical operation
AC 4	24/M	Duodenal ulcer perforation	288.15	4,285.613	0.064	9.15	17.9	7.2	37.1	N 1 and F 0	Surgical operation
AC 5	18/M	HBV carrier	186.76	1,187.912	0.078	9.45	18.7	3.2	30.9	Undetection	Biopsy
AC 6	36/M	HBV carrier	213.07	2,237.512	0.051	8.54	21.1	7.6	32.6	Undetection	Biopsy
AC 7	22/M	HBV carrier	138.72	18.534	0.173	6.86	22.6	6.4	38.7	Undetection	Biopsy
AC 8	26/M	HBV carrier	144.39	88.969	0.249	7.81	9.1	3.1	20.8	Undetection	Biopsy
AC 9	23/M	HBV carrier	119.46	1,084.750	0.094	8.88	8.7	4.2	22.0	N 0 and F 0	Biopsy
AC 10	28/M	HBV carrier	478.03	2,371.143	0.238	8.80	17.4	6.1	32.1	N 1 and F 0	Biopsy
ACH 1	50/M	ACH, hepatic failure	6.12	396.641	0.065	5.34	261.3	114.7	120.8	N 18 and F 2	Liver transplant
ACH 2	46/M	ACH, hepatic failure	3.41	2.693	0.196	2.90	128.3	60.4	4,039.0	N 18 and F 2	Liver transplant
ACH 3	38/M	ACH	291.28	48.509	0.086	4.15	71.1	25.8	917.0	Undetection	Biopsy
ACH 4	22/M	ACH	98.12	33.375	0.079	4.46	19.4	5.1	558.6	Undetection	Biopsy
ACH 5	22/M	ACH	159.12	4.265	0.132	7.61	29.9	14.8	184.5	Undetection	Biopsy
ACH 6	26/M	ACH	148.31	42.274	0.095	7.08	20.9	8.6	323.8	Undetection	Biopsy
ACH 7	21/M	ACH	119.18	731.640	0.067	6.62	9.1	2.2	474.1	Undetection	Biopsy
ACH 8	33/M	ACH	98.14	1,347.302	0.059	8.56	17.9	2.8	130.5	Undetection	Biopsy
ACH 9	32/M	ACH	105.25	564.652	0.112	8.04	12.7	3.8	151.7	Undetection	Biopsy
ACH 10	27/M	ACH	223.97	1,758.515	0.264	9.20	19.6	8.8	532.0	Undetection	Biopsy
ACH 11	28/M	ACH	231.85	7.720	0.102	2.91	23.2	11.4	140.8	N 12 and F 1	Biopsy
ACH 12	40/M	ACH	69.05	152.573	0.050	4.11	173.2	55.9	252.5	N 17 and F 1	Biopsy
ACH 13	20/M	ACH	59.90	0.800	0.068	5.92	35.5	11.8	109.1	N 12 and F 1	Biopsy
ACH 14	39/M	ACH	80.51	560.840	0.052	8.81	25.3	11.3	96.0	N 12 and F 2	Biopsy
Cir 1	36/M	Cirrhosis	153.33	2.775	0.084	6.78	40.6	8.0	43.0	N 8 and F 6	Surgical operation
Cir 2	38/M	Cirrhosis	70.12	8.59	0.183	4.84	41	16.7	66.5	N 8 and F 6	Liver transplant
Cir 3	28/M	Cirrhosis	70.96	417.763	0.108	6.18	57	23.7	96.8	N 8 and F 6	Liver transplant
Cir 4	49/M	Cirrhosis	118.96	7.159	0.063	4.30	57.7	25.9	212.6	N 9 and F 6	Surgical operation
Cir 5	56/M	Cirrhosis	31.87	15.504	0.071	3.85	117.7	56.2	39.0	N 8 and F 6	Liver transplant
Cir 6	43/M	Cirrhosis	145.73	9.49	0.064	6.11	27.8	15.6	60.0	N 5 and F 6	Surgical operation
HCC 1	67/M	Hepatocellular carcinoma	29.87	5.504	0.038	4.53	16.7	6.8	67.4	N 5 and F 2	Surgical operation
HCC 2	46/M	Hepatocellular carcinoma	82.84	11.203	0.115	6.20	30.1	10.0	28.3	N 3 and F 1	Surgical operation
HCC 3	37/M	Hepatocellular carcinoma	147.56	48.483	0.094	7.84	18.6	7.5	89.0	N 5 and F 0	Surgical operation
HCC 4	53/M	Hepatocellular carcinoma	51.34	1.147	0.152	5.88	10.0	4.6	63.0	N 3 and F 1	Surgical operation
HCC 5	56/M	Hepatocellular carcinoma	178.68	1.31	0.052	4.04	24.5	8.4	18.0	N 2 and F 2	Surgical operation
HCC 6	38/F	Hepatocellular carcinoma	181.75	4.312	0.043	6.34	6.7	4.5	69.0	N 3 and F 1	Surgical operation

Protein quantity was estimated by Western blotting in the biopsy subjects AC 5–6 and ACH 3–6, mRNA quantity was detected by real-time quantitative PCR in AC7–8 and ACH 7–10, immunohistochemistry staining was performed in AC 9–10 and ACH 11–14. All three detections, protein quantity, mRNA quantity and immunohistochemistry staining, were performed in the surgical specimens of HD 1–6, AC 1–4, ACH 1–2, Cir 1–6 and HCC 1–6. The necroinflammatory lesions and fibrosis were graded according to the Ishak modified HAI. The maximum possible scores for necroinflammation and fibrosis were 18 and 6, respectively

**Fig. 1:**
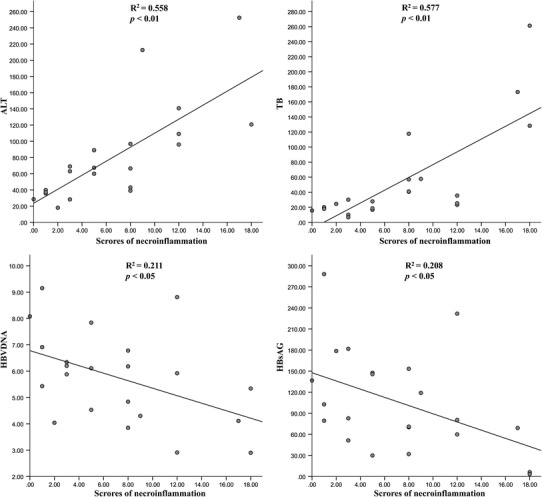
Correlation between intrahepatic scores of necroinflammation and serum ALT, TB, HBsAg or HBV-DNA load. The histological examination of liver tissues was performed in 24 CHB patients, 6 patients in each group. The intrahepatic scores of necroinflammation were graded by the system of Ishak modified HAI. The correlation coefficient between the scores of necroinflammation and ALT, TB, HBsAg or HBV-DNA load was analyzed. The scores of necroinflammation in liver were positively correlated with ALT and TB, but negatively correlated with HBsAg and HBVDNA load in all the CHB patients

On correlation analysis, necroinflammatory scores in liver were positively correlated with ALT and total bilirubin (TB), but negatively correlated with HBsAg and HBV-DNA load in all CHB patients (Fig. 1). The results shown in Fig. 1 suggest that increased ALT and TB in peripheral blood and the severity of necroinflammation in liver were closely interconnected, and that high inflammatory responses partially inhibited HBV replication.

### Identification of CD80 and CD86 proteins in liver

**Fig. 2:**
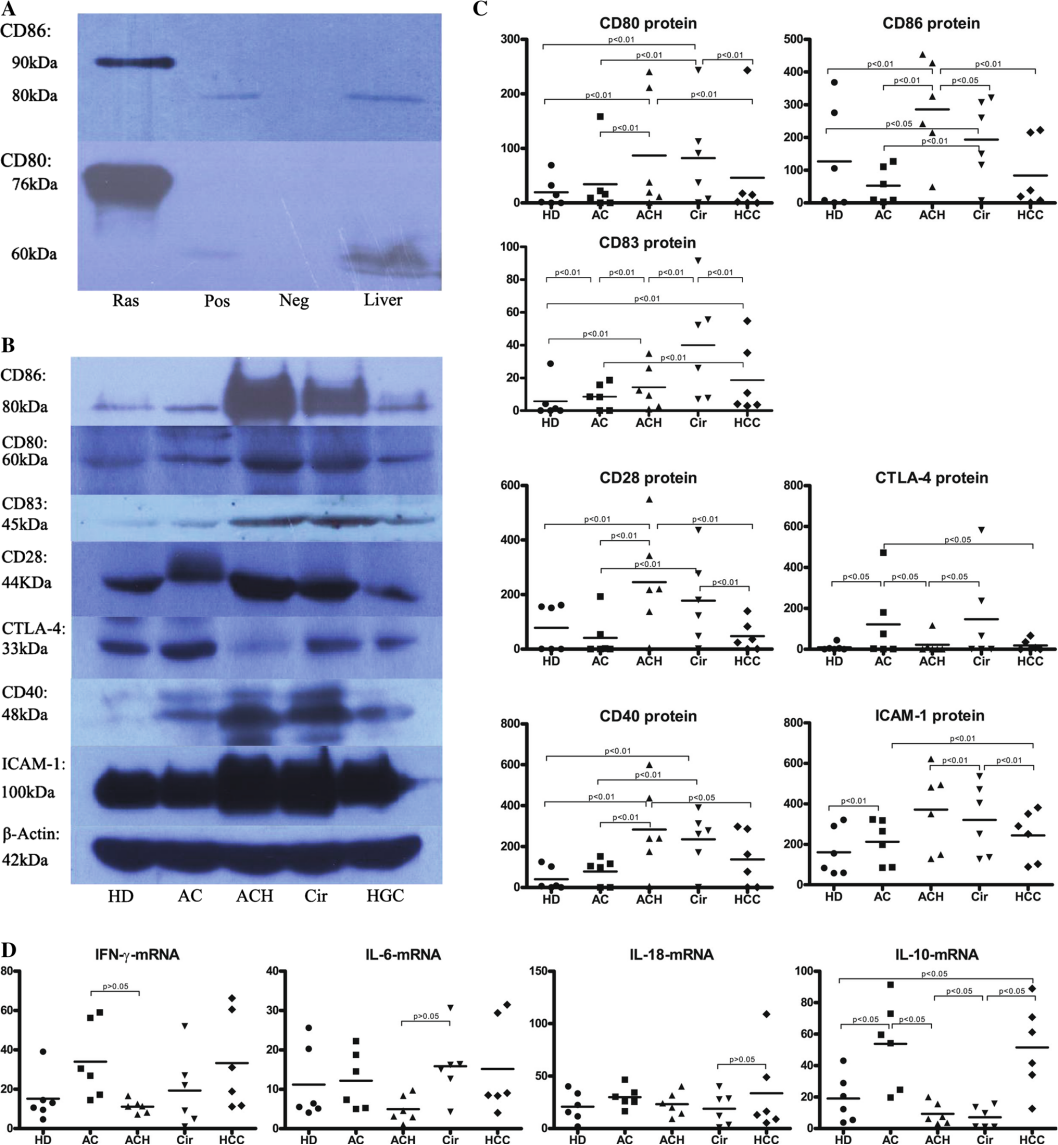
Quantitative detection of costimulatory proteins or cytokine mRNA in liver. **a** Identification of CD80 and CD86 in liver. Western blotting was performed using a specific antibody against human costimulatory protein. Recombinant human CD80 and CD86 proteins, positive control CD80 protein (separate Ramos cell) and CD86 protein (separate Jurkat cell), negative control and liver homogenates of human were applied to determination of CD80 and CD86 in liver tissue. In the same western blotting, recombinant protein (Rec), positive control (Pos), negative control (Neg) and liver homogenates of human (Liver) were subjected to electrophoresis. In the photograph of CD86 protein, the molecular weight of recombinant CD86 protein was 90 kDa, and that of CD86 protein in human liver was 80 kDa. In the photograph of CD80, the molecular weight of recombinant CD80 protein was 76 kDa, and that of CD80 protein in human liver was 60 kDa. **b** Representative western blotting of costimulatory proteins in livers from six separate experiments. Homogenates obtained from the livers in the five groups were subjected to electrophoresis. β-Actin protein served as a protein loading standard. The molecular weight of CD86, CD80, CD83, CD28, CTLA-4, CD40, ICAM-1 and β-actin was 80, 60, 45, 44, 33, 48, 100 and 42 kDa, respectively. The CD80 stains in the ACH or Cir patient were darker than those in the HD, AC or HCC subject. The CD86 stains showed that the stains in the ACH or Cir patient were also darker than those in the HD, AC or HCC subject. In CD83 stains, the photograph showed dark stains of CD83 in the Cir patient. The photograph showed dark stains of CD28 and weak stains of CTLA-4 in the ACH patient. The stains of CTLA-4 in the AC or Cir patient were darker than those in the ACH, HD or HCC patient, whereas the stains in the ACH patient were weaker than those in other subjects. As the CD40 photograph showed, the dark stains of CD40 presented in the ACH or Cir patient. The ICAM-1 stains showed that the stains in the ACH and Cir patients were darker than those in the HD, AC and HCC subjects. **c** Relative quantity of costimulatory proteins in livers of the five groups (*n* = 6, in each group). The images on X-ray membrane were scanned, and the relative quantity of protein was normalized to the protein quantity for each sample using β-actin protein. All the parameters were shown in this figure. The data showed increased CD80, CD86, CD83, CD28 and CD40 and decreased CTLA-4 in the ACH patient, increased CD80, CD86, CD83, CD28, CD40 and CTLA-4 in the Cir patient, increased CTLA-4 and decreased CD80, CD86, CD28 and CD40 in the AC subject, and decreased CD80, CD86, CD28, CD40 and CTLA-4 in the HCC patient. The ICAM-1 levels in the five groups were gradually declined in the order of the ACH group, the Cir group, the HCC group, the AC group and the HD group. **d** Quantitative detection of cytokines mRNA in liver. The relative quantity of the INFγmRNA, IL-6 mRNA, IL-18 mRNA and IL-10 mRNA in liver in all the five groups was detected by real-time quantitative PCR. The data showed unchanged INFγmRNA, IL-6 mRNA and IL18 mRNA in all the five groups, and increased IL-10 mRNA in the AC or HCC patient

Costimulation signals are regulated by costimulatory proteins instead of their mRNA. However, human intrahepatic costimulatory proteins have not previously been detected quantitatively [4–9, 20]. In this study, we tried to identify costimulatory proteins in liver. CD80 and CD86 proteins were identified using the method described in Fig. 2a. The molecular weight of CD86 protein in human liver was 80 kDa, and that of CD80 protein was 60 kDa. In this study, we were the first to identify costimulatory proteins in human liver tissue.

### Increased markers of DC maturation in ACH and Cir patients

**Fig. 3:**
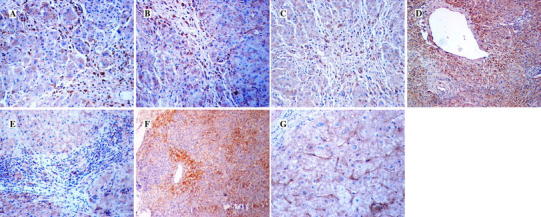
Distribution of intrahepatic costimulatory proteins by immunochemical staining. Immunohistochemical staining was performed using a specific antibody against human costimulatory protein. In this figure, seven costimulation stains in liver of the ACH patient were shown. **a** Distribution of CD80 protein in liver. The hepatocytes were surrounded by CD80^+^ cells and inflammatory cells, ×400 magnification. **b** Distribution of CD86. CD86^+^ cells were localized in the inflammatory zone and they surrounded the hepatocytes, ×400 magnification. **c** Distribution of CD83 protein by immunochemical stains. CD83 stains mainly appeared in the inflammatory cells, and the CD83^+^ cells were localized in the inflammatory-necrotic zone, ×400 magnification. **d** Distribution of CD28 protein in the liver. CD28^+^ cells were enriched in the inflammatory zone, ×100 magnification. **e** CTLA-4 distribution in liver. Few CTLA-4 stains were found in the inflammatory zone in the liver of the ACH patient, ×400 magnification. **f** Distribution CD40 in the liver. CD40 ^+^ cells were enriched in the necrotic zone, and CD40 stains surrounded the hepatocytes, ×100 magnification. **g** ICAM-1 distribution in liver. Most ICAM-1 stains appeared in the hepatic sinus, ×400 magnification

The surface of DCs presents CD80, CD86 and CD83. Matured DCs show signs of increased CD80, CD86 and CD83, resulting in activation of T cells and contributing to immune responses in the liver [7, 11]. Therefore, we analyzed the expression of CD80, CD86 and CD83 and observed the localization of CD80^+^, CD86^+^ and CD83^+^ cells in liver in order to explore the functional state of DCs. First, we quantitatively detected the levels of CD80, CD86 and CD83 in liver by Western blotting. Figure 2b, c show that CD80 protein levels in ACH and Cir patients were higher than those in ACs, HDs or HCC patients (*p* < 0.05), that CD86 protein levels in ACH patients were higher than those in ACs, HDs, or HCC or Cir patients, that CD86 protein levels in Cir patients were higher than those in HDs or ACs (*p* < 0.05), that CD83 protein levels in Cir patients were higher than those in HDs, ACs, or ACH or HCC patients (*p* < 0.05), and that CD83 protein levels in ACH or HCC patients were also higher than those in HDs or ACs (*p* < 0.05). The distribution of CD80, CD83 and CD86 proteins in liver was subsequently explored by immunohistochemistry. CD80^+^, CD83^+^ or CD86^+^ cells appeared in the inflammatory zone in the liver tissue of ACH (Fig. 3a–c) or Cir patients, but few positive cells were identified in ACs, HDs or HCC patients. ACH or Cir patients displayed increased CD80, CD83 and CD86. These results implied that matured CD80^+^, CD83^+^ and CD86^+^ DCs participated in inflammatory responses in the ACH or Cir patients.

### CD28^+^ cells participate in necroinflammation in ACH or Cir patients, and increased CTLA-4 contributes to immune tolerance in ACs

CD28 and CTLA-4, acting as downstream molecules of CD80 and CD86, are attached to the surface of T cells. The CD80, CD86/CD28 pathway provides critical costimulatory signals for naive T cells. Conversely, the CD80, CD86/CTLA-4 pathway acts as a key negative regulator of CD28-dependent T cells [21]. To evaluate the role of costimulation on T cells, we quantitatively detected CD28 and CTLA-4 in liver. As shown in Fig. 2b, c, CD28 in ACH patients was significantly higher than in ACs, HDs or HCC patients (*p* < 0.05), and CD28 in Cir patients was higher than in ACs or HCC patients (*p* < 0.05). CTLA-4 in ACs was higher than in HDs or ACH or HCC patients (*p* < 0.05) and CTLA-4 in Cir patients was higher than in ACH patients (*p* < 0.05; Fig. 2b, c). As shown in Fig. 3d, e, CD28^+^ cells were localized in the inflammatory zone, but low levels of CTLA-4 staining were found in the inflammatory zone in ACH patienst. CD28^+^ cells and CTLA-4 staining were both markedly displayed in Cir patients. These data showed that CD28 increased and CTLA-4 decreased in ACH patients, that CD28 decreased and CTLA-4 increased in ACs, and that both CD28 and CTLA-4 increased in Cir patients. The above results suggest that CD28^+^ cells participate in inflammation in ACH and Cir patients, and that increased CTLA-4 contributed to immune tolerance in ACs.

### CD40^+^ cells and ICAM-1 both participate in necroinflammation in ACH and Cir patients

Functional T cells induce activation of B cells and other immune cells, and cause secretion of inflammatory cytokines. CD40 is expressed on the surface of immune cells or non-immune cells such as B cells, monocytes, endothelial cells, epithelial cells, mesenchymal cells, platelets and malignant tumor cells. Therefore, increased CD40 implies that various immune responses occur in liver disease [22]. ICAM-1 participates in adhesion between various immune cells. Furthermore, both CD40 and ICAM-1 contribute to the inflammatory reaction and fibrosis generation in progressive liver disease [23]. Therefore, we quantitatively detected the levels of CD40 and ICAM-1. Fig. 2b, c shows that CD40 protein levels in ACH patients were higher than in HDs or ACs or HCC patients (*p* < 0.05), and CD40 in Cir patients was higher than in HDs or ACs (*p* < 0.05). Figure 2c shows a gradual decrease of ICAM-1 in the five groups in following order: ACH, Cir and HCC patients, ACs and HDs. Increased CD40 and ICAM-1 were present in ACH and Cir patients. As shown in Fig. 3f, g, CD40 staining and CD40^+^ cells were enriched in the necroinflammatory zone, and ICAM-1 protein was found in the hepatic sinus. CD40 and ICAM-1 were strongly observed in the liver tissue of ACH and Cir patient. These results implied that CD40^+^ cells and ICAM-1 both contributed to necroinflammation in ACH and Cir patients.

### Increased IL-10 mRNA in liver of AC and HCC patients

Cytokines are divided into inflammatory cytokines and inhibitory cytokines. INFγ, IL-6 and IL-18 participate in acute inflammatory responses in liver. More importantly, IL 18 expression is mainly located in the liver. Conversely, IL-10 is an inhibitory cytokine and generally contributes to immune suppression. Soluble cytokines are expressed by immune cells and immediately released into the blood. The concentration of cytokine proteins in liver is unstable, but cytokine mRNA in tissue is stable because cytokine mRNA is not released into the blood [18]. We therefore sought cytokine mRNA in liver. Figure 2e shows that there were no differences in INF-γ mRNA, IL-6 mRNA and IL-18 mRNA between any of the five groups (*p* > 0.05). However, IL-10 mRNA levels in ACs and HCC patients were higher in Cir patients (*p* < 0.05), and IL-10 mRNA in ACs was higher than in ACH patients (*p* < 0.05). These results showed that INF-γ mRNA, IL-6 mRNA and IL-18 mRNA in all five groups stayed unchanged, but that IL-10 mRNA increased in ACs and HCC patients.

### Correlations between ALT and costimulatory proteins and between costimulatory proteins in four groups

To identify the effects of costimulation on CHB, we assessed the correlation coefficient between intrahepatic costimulation profiles and serum ALT (see Table 4). The ACH patients showed positive correlations between ALT and inflammatory costimulatory proteins CD80, CD83, CD28, CD40 and ICAM-1 (*p* < 0.05), but negative correlations between CTLA-4 and inflammatory costimulatory proteins CD80, CD86, CD83, CD28, CD40 and ICAM-1 (*p* < 0.05). In AC patients, CTLA-4 was negatively correlated with ALT, CD83, CD40 and ICAM-1 (*p* < 0.05), and IL-10 mRNA was negatively correlated with ALT (*p* < 0.05). Interestingly, ALT was positively correlated with CD80, CD86, CD83, CD28, CD40 and ICAM-1 (*p* < 0.05), although ALT was below the upper limit of normal in all six ACs. A positive correlation between ALT and CD80 (*p* < 0.05) was present in the Cir patients. In addition, a negative correlation between ALT and IL-10 mRNA and a positive correlation between CTLA-4 and CD83 (*p* < 0.05) were displayed in the HCC patients. These results indicate that both increased inflammatory costimulatory proteins and decreased inhibitory costimulatory proteins participated in hepatitis activation in ACH patients, and that inhibitory costimulatory proteins CTLA-4 and IL-10 contributed to immune tolerance and maintained normal hepatic function in ACs.

**Table 4:** Pairwise correlation between ALT and costimulatory proteins, or between CTLA-4 and other costimulatory proteins

	ALT	CD80	CD86	CD83	CD28	CTLA-4	CD40	ICAM-1	IL-10 mRNA
ALT									
AC group	*R*: 1	0.756*	0.878*	0.889*	0.737*	−0.855*	0.914*	0.922*	−0.909*
	*p*:	0.041	0.01	0.009	0.047	0.015	0.005	0.004	0.006
ACH group	*R*: 1	0.807*	0.684	0.859*	0.890*	−0.643	0.850*	0.738*	−0.528
	*p*:	0.026	0.067	0.014	0.009	0.084	0.016	0.047	0.141
Cir group	*R*: 1	0.972*	0.028	0.200	−0.080	−0.522	0.311	−0.256	0.678
	*p*:	0.001	0.958	0.704	0.881	0.288	0.549	0.625	0.069
HCC group	*R*: 1	0.115	0.577	0.301	0.614	0.267	-0.432	−0.586	−0.752*
	*p*:	0.826	0.231	0.562	0.194	0.609	0.393	0.222	0.042
CTLA-4									
AC group	*R*: –0.855	−0.426	−0.675	−0.776*	−0.421	1	−0.842*	−0.831*	
	*p: 0.015	0.200	0.071	0.035	0.203		0.018	0.02	
ACH group	*R*: –0.643	−0.821*	−0.961*	−0.903*	−0.908*	1	−0.925*	−0.948*	
	*p*: 0.084	0.022	0.001	0.007	0.006		0.004	0.002	
Cir group	*R*: −0.522	−0.646	−0.682	−0.341	0.585	1	0.193	−0.590	
	*p*: 0.288	0.166	0.135	0.508	0.222		0.714	0.217	
HCC group	*R*: 0.267	−0.339	0.423	0.978*	0.592	1	−0.559	0.350	
	*p*: 0.609	0.511	0.404	0.001	0.216		0.249	0.496	

* *p* < 0.05

## Discussion

Costimulatory factors participate in immune responses to HBV components, and induce maturation of DCs, activation of T cells and other immune cells, and secretion of cytokines. Overall, the intrahepatic costimulation profile represents opposing immunocompetences, either inflammatory or suppressive immune responses, which characterize the actual immunopathophysiology of CHB [7, 22, 24]. Our data showed decreased CTLA-4 and IL-10 and increased CD80, CD86, CD83, CD28, CD40 and ICAM-1 in the liver of ACH patients. In contrast, a special costimulatory expression, namely increased inhibitory costimulatory factors and decreased inflammatory costimulatory proteins, presented in the ACs. However, the costimulation profiles in the Cir and HCC patients were confusing. An inconsistent expression of costimulatory factors, namely increased expression of both inhibitory and inflammatory costimulatory factors, occurred in the Cir patients. In contrast, decreased expression of both inhibitory and inflammatory costimulatory factors was present in the HCC patients. The CHB patients presented various costimulation profiles in liver.

Previous investigations and the present study both demonstrated that intrahepatic costimulation profiles contributed to immunopathogenesis in CHB patients. Chen et al. and Zou et al. [25, 26] investigated intrahepatic IL-10, INF-γ or programmed death 1 (PD-1) receptors in CHB patients using immunohistochemistry and assessed the correlation between the immunohistochemical intensity in liver and ALT in serum. Stoop et al. [14] observed intrahepatic regulatory T cells using flow cytometry and evaluated the role of these cells using correlation analysis. Using immunohistochemistry and histology, we observed the distribution of costimulatory proteins and necroinflammation scores in liver to be consistent with previous studies. The cells with positive inflammatory costimulatory proteins were localized in the necroinflammatory zone in the liver of ACH and Cir patients. We therefore considered that various inflammatory cells participate in the immunopathogenesis of CHB by regulating costimulatory proteins. In addition, intrahepatic costimulation profiles were quantitatively detected using Western blotting, and the relationship between the intrahepatic costimulation profile and serum ALT was assessed in the present study. The results demonstrated that increased inflammatory costimulation and decreased inhibitory costimulation both contributed to intrahepatic necroinflammation and caused abnormal hepatic function in ACH patients. In contrast, increased inhibitory costimulation and decreased inflammatory costimulation in ACs resulted in no intrahepatic necroinflammation and in normal serum ALT, representing immune tolerance. However, the costimulation profiles in the Cir and HCC patients were puzzling, and we were not able to explain the associations in the costimulation profiles. Our data further revealed that the intrahepatic costimulation profiles, instead of a single costimulatory factor, contributed to immunopathogenesis in CHB patients and resulted in multiple abnormalities in immune response pathways, including DC maturation, immune cell activation and cytokine secretion.

In Asia, the majority of HBV infection is acquired perinatally or in early childhood; a large number of patients are therefore in the immune-tolerant phase [2]. Because of the lack of a suitable animal model for the investigation of the immune state of CHB patients, almost nothing is known about the mechanism of immune tolerance and the differences between immune tolerance and immune injury. The immune tolerance of CHB patien is still a mysterious phenomenon. In the present study, the differences between costimulation profiles in the liver of ACs and those of the ACH, Cir and HCC patients were shown for the first time, revealing some of the characteristics of immune tolerance.

Notably, opposite CTLA-4 expressions were present in ACs and HCC patients, namely increased CTLA-4 in the ACs and decreased CTLA-4 in the HCC patients. CTLA-4 and PD-1 inhibit T-cell activation through different pathways: PD-1 inhibits CD28-mediated activation of phosphatidylinositol 3-kinase (PI3K), whereas CTLA-4 activates type II serine/threonine phosphatase PP2A. Both CTLA-4 and PD-1 limit T-cell activation [27]. In a study by Nakamoto et al., CD28 was highly expressed in intrahepatic PD-1^+^CTLA-4^+^CD8 T cells compared to CTLA-4^−^ CD8 T cells, and increased CD28 is a marker of T-cell activation. By competing with CD28 for CD80 and CD86, CTLA-4 may inhibit CD28^+^ T-cell activation by specific signaling pathways. Interestingly, CTLA-4 may play different roles in various signaling pathways in viral infections, such as lymphocytic choriomeningitis virus, human immunodeficiency virus and hepatitis C virus [16]. The above studies lead us to speculate that these opposing CTLA-4 expressions in ACs and HCC patients might result from various CTLA-4 signaling pathways for immune tolerance of T cells or generation of HCC. However, the mechanism remains unknown.

The present study was an observational investigation of human liver. We would like to improve the work by investigating further cases or by performing more techniques. However, ethical principles in the clinical study limited the acquisition of human tissue. In previous clinical investigations, a single immunological technique was applied to exploring intrahepatic immunopathogenesis in CHB patients, such as immunohistochemistry in studies by Chen et al. and Zou et al. [25, 26] or flow cytometry in Stoop et al. [14]. However, immunohistochemical pathologists, such as Becker and Taylor [28], believe that immunohistochemistry-based methods retain morphological data but prove difficult to quantify, and that Western blotting may be used for quantitative detection of protein in tissue. In a study by Philips et al., both Western blotting and immunohistochemistry were used to investigate enzyme proteins in the human bladder [29]. According to Becker’s strategy and Phillips’ measurements, we applied two techniques, immunohistochemistry and Western blotting, to explore the effects of costimulatory proteins in liver. A limit of 6 liver specimens from each group was used in the present study, which followed both ethical principles and statistical principles in an observational investigation of human tissue. In addition, we wish to further explore the mechanism of immune tolerance and immune injury on costimulation using analysis of signal pathways. However, this intent is merely a wish because a suitable animal model with the whole disease spectrum of CHB has not been established and perfect human specimens for investigation of signal pathways cannot be obtained. For the first time, the profiles of intrahepatic costimulation in CHB patients were investigated here, which revealed the natural immune status of the patients. A prominent feature of the present study was that the human liver with the whole disease spectrum of CHB, instead of cell culture in vitro or intervening analysis in an animal model, was applied to investigate immunopathophysiology. Despite the present study being preliminary, the findings are groundbreaking in the field of chronic HBV infection.

In conclusion, various profiles of costimulation are present in the liver of CHB patients. Costimulation participates in immune responses in liver and plays important roles in immune tolerance in ACs, in immune injury in ACH patients and in immune abnormalities in Cir and HCC patients. The multiple abnormalities in the immune response create the immunopathogenesis of CHB patients.

## Electronic supplementary material

Below is the link to the electronic supplementary material.

Supplementary material 1 (DOC 41 kb)

## Abbreviations

ACs: Asymptomatic carriers
ACH: Active chronic hepatitis
ALT: Alanime aminotransferase
CHB: Chronic HBV infection
Cir: Cirrhosis
CTLA-4: Cytotoxic *T*-lymphocyte antigen 4
DCs: Dendritic cells
HBV: Hepatitis B virus
HCC: Hepatocellular carcinoma
HD: Healthy donors
ICAM-1: Intercellular adhesion molecule-1
PD1: Programmed cell death -1
TB: Total bilirubin
