# Supplementary material for: Effects of costimulation on intrahepatic immunopathogenesis in patients with chronic HBV infection
Source: Inflamm Res. 2013 Dec 14;63(3):217–29. doi: 10.1007/s00011-013-0691-3 (PMC3921453; doi:10.1007/s00011-013-0691-3)
Supplement: Supplementary file 1 — Supplementary material 1 (DOC 41 kb) [file 11_2013_691_MOESM1_ESM.doc]

Explanation for research strategy and experiment methods

In accordance with statistical rules, a sufficient sample of tissue is required in clinical investigation and animal experiment. The present study was an observed investigation of the human liver. We would like to improve the work by investigating more cases and by performing effective techniques. However, the ethical principles in the clinical study limited the acquisition of human tissue. It is more difficult that the research design is suitable for both the ethical principles in clinical study and the statistical rules on the quantitative analysis. Therefore, a series of preliminary experiments in the present study were firstly carried out for determining the method of quantitative detection. Then, sample size was determined based on previously published papers, our earlier works and the preliminary experiments in the present study.

Selection of quantitative detection for costimulatory protein in liver by western blotting instead of stores of protein stains by immunohistochemistry

In previous studies administrated by clinicians or clinical investigators, the percentage of positive cells with costimulatory proteins or the scores of positive stains with costimulatoty proteins in human’s tissue between different groups were compared for exploring the pathogenesis in liver disease on costimulatory proteins [1-3]. Scores of costimulatory protein stains in liver by immunohistochemistry, a semi-quantitative detection, was performed in Chen’s and Zou’s papers. The percentage of cells with costimulatory protein was detected by flow cytometry in Stoop’s study. However, immunohistochemistry pathologists, such as Becher K and Taylor CR, believe that immunohistochemistry based methods retain morphological data but have proven difficult to quantify, and that western blot may be applied to quantitative detection of protein in froze tissue, even in formalin-fixed paraffin-embedded sections [4]. Therefore, many researchers have applied western blot to identifying and quantitative detecting proteins, instead of immunohistochemistry. Their papers have published in the professional journal of histochemistry, such as Acta Histochem Cytochem [5]. In accordance with statistical principles, the sample size in quantitative analysis is less than that in the semi-quantitative detection. Using quantitative detection of protein instead of semi-quantitative of protein stains scores, 6 samples, even 3 samples, may be suitable for clinic investigation of human tissue or animal experiment [6-11]. Therefore, in our preliminary experiment we firstly improved the technique of western blot that is suitable for quantitative detection of intrahepatic costimulatory protein (in the section of ‘Western blotting of costimulatory protein in liver’ in Materials and methods). Then an investigating program, namely distribution of protein in tissue by immunohistochemistry, quantitative detection of protein by western blotting, quantitative detection of cytokine mRNA by real-time quantitative PCR and correlation analysis among ALT, scores of necroinflammation, cytokine mRNA and costimulation proteins, was determined.

Determination of liver sample size in the present study

Before the present research, we referred to sample size in animal experiments or clinic investigations in the papers published. The number of sample in the animal investigations were 3 or 4 in studied groups [6, 7], and even less than 3 animals [8]; while in our previous study for observing physiological function, 5-6 macaques or rabbits were used for each group [9, 10]. In the study on edotoxin tolerance by Western blotting, we used mice in groups of six too [11]; and we obtained valid data which were accepted for publication. In an observed research of human liver, the sample size was critically limited because of ethical limitation. Coffin CS used liver samples of humans with transplanted liver to observe the resistant mutations of entecavir, lamivudine, adefovir, tenofovir and emtricitabine in liver, only nine cases [12].

Prior to this study, we asked statisticians for advice and they suggested that 6 liver specimens in studied groups be sufficient and acceptable by quantitative detection of intrahepatic costimulation proteins. Our preliminary experiment on CD80 and CD86 showed significant difference in groups of six samples. Then we designed the present research on the human liver, limit of 6 liver specimens for each group, and got the design approved by the Committee for the Ethical Care of Southeast University, which accorded with both ethical and statistical principles in the clinic investigation.

Liver sample, unlike blood sample, cannot be obtained in large numbers; and compared with the samples in all the published papers in the same field we could refer to, the sample size of our study was the largest. More importantly, the samples obtained from AC and HD subjects were especially difficult to collect because of the sensitive ethical issue, which is probably why there have been no studies in this field.

Reference:

1. J. Chen, X.M. Wang, X.J. Wu, Y. Wang, H. Zhao, B. Shen, G.Q. Wang, Intrahepatic levels of PD-1/PD-L correlate with liver inflammation in chronic hepatitis B, Inflamm Res 2011 60 47-53.

2. Z. Zou, B. Li, D. Xu, Z. Zhang, J.M. Zhao, G. Zhou, Y. Sun, L. Huang, J. Fu, Y. Yang, L. Jin, W. Zhang, J. Zhao, Y. Sun, S. Xin, F.S. Wang, Imbalanced intrahepatic cytokine expression of interferon-gamma, tumor necrosis factor-alpha, and interleukin-10 in patients with acute-on-chronic liver failure associated with hepatitis B virus infection, J Clin Gastroenterol 43(2009) 182-190.

3. J.N. Stoop, M.A. Claassen, A.M. Woltman, R.S. Binda, E.J. Kuipers, H.L. Janssen, R.G. van der Molen, A. Boonstra, Intrahepatic regulatory T cells are phenotypically distinct from their peripheral counterparts in chronic HBV patients, Clin Immunol 129(2008) 419-427.

4. [Becker KF](http://www.ncbi.nlm.nih.gov/pubmed?term=Becker KF%5BAuthor%5D&cauthor=true&cauthor_uid=20861790), [Taylor CR](http://www.ncbi.nlm.nih.gov/pubmed?term=Taylor CR%5BAuthor%5D&cauthor=true&cauthor_uid=20861790). "Liquid morphology": Immunochemical analysis of proteins extracted from formalin-fixed paraffin-embedded tissues: Combining proteomics with immunohistochemistry. [Appl Immunohistochem Mol Morphol.](http://www.ncbi.nlm.nih.gov/pubmed?term=Karl-Friedrich Becker%2C Clive R. Taylor%2C  D.Philw" \l "%23) 2011 Jan;19(1):1-9.

5. [Philips BJ](http://www.ncbi.nlm.nih.gov/pubmed?term=Philips BJ%5BAuthor%5D&cauthor=true&cauthor_uid=18227726), [Dhir R](http://www.ncbi.nlm.nih.gov/pubmed?term=Dhir R%5BAuthor%5D&cauthor=true&cauthor_uid=18227726), [Hutzley J](http://www.ncbi.nlm.nih.gov/pubmed?term=Hutzley J%5BAuthor%5D&cauthor=true&cauthor_uid=18227726), [Sen M](http://www.ncbi.nlm.nih.gov/pubmed?term=Sen M%5BAuthor%5D&cauthor=true&cauthor_uid=18227726), [Kelavkar UP](http://www.ncbi.nlm.nih.gov/pubmed?term=Kelavkar UP%5BAuthor%5D&cauthor=true&cauthor_uid=18227726). Polyunsaturated fatty acid metabolizing 15-Lipoxygenase-1 (15-LO-1) expression in normal and tumorigenic human bladder tissues. [Appl Immunohistochem Mol Morphol.](http://www.ncbi.nlm.nih.gov/pubmed?term=Brian J. Philips%2C Rajiv Dhir%2Cw Justin Hutzley%2CMalabika Sen" \l "%23) 2008;16(2):159-64.

6．Chan C, Li L, McCAll CE, Yoza BK. Endotoxic tolerance disrupts chromatin remodeling and NF-κB transactivation at the IL-1β promoter. J Immunol. 2005; 175: 461-68.

7．Fan J, Kapus A, Marsden PA, Li YH, Oreopoulos G, Marshall JC et al. Regulation of Toll-like receptor 4 expression in the lung following hemorrhagic shock and lipopolysaccharide. J Immunol. 2002; 168: 5252-9.

8．Didierlaurent A, Brissoni B, Velin D, Aebi N, Tardivel A, Käslin E, Sirard JC, Angelov G, Tschopp J, Burns K.Tollip regulates proinflammatory responses to interleukin-1 and lipopolysaccharide. Mol Cell Biol. 2006 Feb;26(3):735-42.

9. Yin GQ, Qiu HB, Du KH, Tang JQ, Lu CP, Fang ZX Endotoxic shock model with fluid resuscitation in Macaca mulatta. Lab Anim. 2005 Jul;39(3):269-79.

10. Zhu XY, Gu XJ, Chang JB, Zhang Y, Feng YH, Yin GQ. Changes of blood-cerebrospinal fluid barrier in rabbits with diabetic ketoacidosis. Zhongguo Wei Zhong Bing Ji Jiu Yi Xue. 2004 Mar;16(3):175-8.

11. B. Zhong, H.Y. Ma, Q. Yang, F.R. Gu, G.Q. Yin, C.M. Xia, Decrease in toll-like receptors 2 and 4 in the spleen of mouse with endotoxic tolerance, Inflamm Res 57(2008) 252-259.

12. [Coffin CS](http://www.ncbi.nlm.nih.gov/pubmed?term=Coffin CS%5BAuthor%5D&cauthor=true&cauthor_uid=20626626), [Mulrooney-Cousins PM](http://www.ncbi.nlm.nih.gov/pubmed?term=Mulrooney-Cousins PM%5BAuthor%5D&cauthor=true&cauthor_uid=20626626), [Peters MG](http://www.ncbi.nlm.nih.gov/pubmed?term=Peters MG%5BAuthor%5D&cauthor=true&cauthor_uid=20626626), [van Marle G](http://www.ncbi.nlm.nih.gov/pubmed?term=van Marle G%5BAuthor%5D&cauthor=true&cauthor_uid=20626626), [Roberts JP](http://www.ncbi.nlm.nih.gov/pubmed?term=Roberts JP%5BAuthor%5D&cauthor=true&cauthor_uid=20626626), [Michalak TI](http://www.ncbi.nlm.nih.gov/pubmed?term=Michalak TI%5BAuthor%5D&cauthor=true&cauthor_uid=20626626), [Terrault NA](http://www.ncbi.nlm.nih.gov/pubmed?term=Terrault NA%5BAuthor%5D&cauthor=true&cauthor_uid=20626626). Molecular characterization of intrahepatic and extrahepatic hepatitis B virus (HBV) reservoirs in patients on suppressive antiviral therapy. [J Viral Hepat.](http://www.ncbi.nlm.nih.gov/pubmed/20626626#%23) 2011 Jun;18(6):415-23.
